# The effectiveness of omega‐3 fatty acids on health outcomes in women with breast cancer: A systematic review

**DOI:** 10.1002/fsn3.3409

**Published:** 2023-05-22

**Authors:** Shirin Osouli‐Tabrizi, Amir Mehdizadeh, Mina Naghdi, Zohreh Sanaat, Nafiseh Vahed, Azizeh Farshbaf‐Khalili

**Affiliations:** ^1^ Department of Midwifery, Faculty of Nursing and Midwifery Tabriz University of Medical Sciences Tabriz Iran; ^2^ Hematology and Oncology Research Center Tabriz University of Medical Sciences Tabriz Iran; ^3^ Student Research Committee, Department of Midwifery, Faculty of Nursing and Midwifery Tabriz University of Medical Sciences Tabriz Iran; ^4^ Research Center for Evidence‐based Medicine, Iranian EBM Centre: A Joanna Briggs Institute (JBI) Center of Excellence Tabriz University of Medical Sciences Tabriz Iran; ^5^ Physical Medicine and Rehabilitation Research Center, Aging Research Institute Tabriz University of Medical Sciences Tabriz Iran

**Keywords:** adjuvant therapy, breast cancer, health, Omega‐3 fatty acids, systematic review

## Abstract

This study aimed to systematically evaluate the impact of omega‐3 fatty acids on the health outcomes of women with breast cancer in electronic databases (PubMed, Scopus, ProQuest, Web of Science, and Cochrane Library) for interventional studies. The risk of bias and the quality of the included articles were assessed by Cochrane Collaboration Handbook guidance. The statistical analyses were not conducted because of the heterogeneity of the included studies. Of 3676 identified articles, 11 articles were included in this study. The majority of the included studies were not of high quality. Median progression time and overall survival significantly improved. Additionally, surgical site healing complications and infection rates decreased. There was a significant decrease in perceived stress, sleep disturbance, depression, pain, joint stiffness, and fatigue throughout the intervention. Moreover, omega‐3 fatty acids consumption significantly increased the total serum omega‐3, EPA, and DHA, and decreased the omega‐6: omega‐3 ratio, total leukocytes, lymphocytes, leptin, and CRP, accordingly. Mild gastrointestinal symptoms were reported in only two studies without clinically relevant adverse events. Omega‐3 fatty acids may cause improvement in physical, mental, and some inflammatory and metabolic indices during treatment or posttreatment course of breast cancer patients. Due to the possibility of free radical formation, omega‐3 FAs supplementation and consumption must be done very carefully.

## Graphical abstract

This study aimed to evaluate systematically the impact of omega‐3 fatty acids supplementation on the health outcomes of women with breast cancer in the electronic databases for interventional studies.

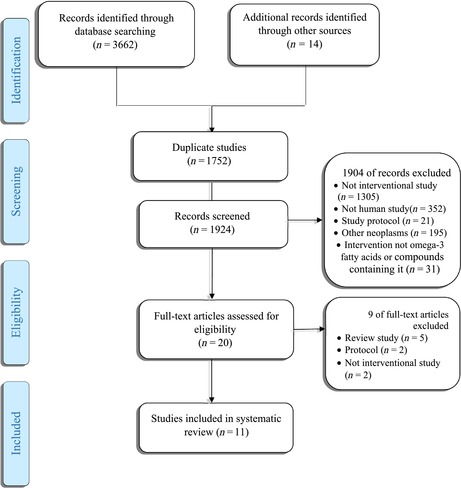

## 1 INTRODUCTION

Breast cancer (BC) is the most common cancer among women and is the fifth leading cause of death due to cancer. In 2020, about 2.3 million new cases and 658,000 deaths due to BC were recorded (Sung et al., 2021). In general, BC is a multifactorial disease. The main risk factors of BC include genetic factors, family history of BC in the first relatives, past history of ovarian cancer, lifestyle, not marrying, and late age menopausal (Hamdi‐Cherif et al., 2020; Rojas & Stuckey, 2016). Mammography is one screening method for BC diagnosis, and this screening method has been proven to reduce the mortality rate in women (Elmore et al., 2005). The most common therapeutic strategies for BC are chemotherapy, surgery, radiotherapy, immunotherapy, hormone therapy, and gene therapy (Arruebo et al., 2011). However, the toxicity of antineoplastic treatments affects the patient's quality of life. The therapeutic intervention also causes short‐term and long‐term side effects such as fatigue, dizziness, constipation, appetite decrease, nausea, vomiting, and cardiotoxicity (Partridge et al., 2001; Shapiro & Recht, 2001).

In this regard, recent studies have reported the effectiveness of complementary medicine on the improvement of treatment results in different cancers with minimal side effects. Hence, co‐adjuvant therapies are of profound interest (Gerber et al., 2006; Hübner et al., 2021; Zhu et al., 2016).

Omega‐3 fatty acids (omega‐3 FAs) supplementation is considered an adjunctive treatment to reduce toxicity and improve disease outcomes (Laviano et al., 2013). Omega‐3 FAs play an important role in cell membrane structure, fluidity, and cell signaling (Calder, 2011). Some studies have shown that dietary omega‐3 polyunsaturated fatty acids (PUFAs) reduce the development of cancer, including BC (Bartsch et al., 1999; Chen et al., 2007; Turk & Chapkin, 2013). It has been reported that the aberrant expression of enhancer of zest homologue 2 (EZH2) is associated with metastasis and poor outcomes in cancer patients. In this regard, unsaturated omega‐3 FAs consumption causes EZH2 regulation in BC (Dimri et al., 2009). A chronic inflammatory status leads to tumor initiation, growth, and metastasis (Hammad et al., 2021). Omega‐3 FAs reduce cytokine production, decrease cell proliferation and tumor growth, as well as increase the apoptosis process. Some studies have also shown that omega‐3 FAs play an essential role in anti‐inflammatory actions (Calder, 2013; Mori & Beilin, 2004; Schley et al., 2005; Yenipazar & Şahin‐Yeşilçubuk, 2022). Moreover, omega‐3 supplementation increases the skeletal muscle index, energy balance, and overall survival (OS), while reducing xerostomia, inflammation, and toxicity of chemotherapy. In the Ambrosone et al. (2020) study, the use of omega‐3 FAs both before and during chemotherapy was associated with disability‐free survival (DFS) but no significant improvement was observed regarding OS. Some studies have also shown that omega‐3 FAs have a positive effect on the immune system (Bird et al., 2021; Darwito et al., 2019; de la Rosa Oliva et al., 2019; Ma et al., 2021).

Inflammation in cancer patients may be associated with complications such as pain and depression. Depression is another common problem affecting 8%–24% of cancer patients. It is assumed that the depletion of omega‐3 FAs in cell membranes is of etiological importance in depression. Some studies supported the antidepressant properties of omega‐3 FAs consumption (Chiu et al., 2003; Krebber et al., 2014; Maes & Smith, 1998). Peripheral neuropathy is a common side effect of many chemotherapeutic patients. Additionally, omega‐3 FAs may be effective in the prevention of paclitaxel‐induced neuropathy (PIPN) in women with BC who undergo chemotherapy and increase the quality of life in these patients (Ghoreishi et al., 2012).

Although omega‐3 supplements have no side effects during chemotherapy and can improve chemotherapy outcomes when highly incorporated (Bougnoux et al., 2009), another study reported mild gastrointestinal tract (GIT) discomfort (Martínez et al., 2019). It should be noted that when prescribing omega‐3 PUFAs, side effects should be considered. In this regard, the results of a study showed no short‐term complications after omega‐3 PUFAs consumption; however, long‐term PUFA supplementation may be associated with an increased risk of cancer, possibly due to their oxidation products or added antioxidants (Lange et al., 2019). On the other hand, the source of omega‐3 FAs may be important in acquiring positive outcomes. The results of a study showed a reduced risk of recurrence after colon cancer diagnosis following omega‐3 PUFAs following fish consumption and not supplements (Van Blarigan et al., 2018).

Despite the numerous benefits of omega‐3 FAs, previous systematic reviews have been limited in the lack of comprehensive literature on the health outcomes of women with BC. Therefore, the aim of the current systematic review was to summarize the effect of omega‐3 FAs supplementation on physical and psychological outcomes, as well as metabolic indices in women suffering from BC.

## 2 MATERIALS AND METHODS

This systematic review was performed according to the guidelines of the Cochrane Handbook for systematic reviews of interventions (Higgins et al., 2022) and Preferred Reporting Items for Systematic Reviews and Meta‐Analyses (PRISMA; Shamseer et al., 2015). In this study, all the interventional studies consisted of randomized controlled trials (RCTs), and quasi‐ and semi‐experimental articles that investigated the effect of supplementation with omega‐3 FAs on the health outcomes of women with BC were studied.

### 2.1 Inclusion criteria

In this survey, the PICOS criteria including participants, intervention, comparison, outcomes, and study design were used. The participants included women with BC under medical treatment or in received‐treatment follow‐up state. The intervention comprised the use of omega‐3 FAs supplements or consuming foods enriched with omega‐3 FAs sources. The comparison included any control group such as a placebo, any other known supplements, or without intervention. Outcomes included any health outcomes of supplementation such as physical, mental, and metabolic outcomes. The study design included RCTs, quasi, and semi‐experimental studies.

Omega‐3 FAs could be administered during any medical treatment such as surgery, chemotherapy, radiation, and their combination, or during the follow‐up of BC survivors' medical treatment. All the related articles on BC patients at any stage were included. Omega‐3 FAs supplements in any form, fish oil, docosahexaenoic acid (DHA), EPA, and alpha‐linolenic acid (ALA), etc., at any dose, by any route (oral or enteral), at any duration and dose interval, solely or in combination with other nutrients, were included in this review. Dietary interventions through consuming foods enriched by omega‐3 FAs sources were also incorporated. There was no age restriction.

### 2.2 Exclusion criteria

Review articles, animal studies, cellular and molecular studies, study protocols, cross‐sectional studies, cohorts, case–control, case reports, case series, letters to the editor, and editorials were excluded.

### 2.3 The strategy of articles search and study selection

A comprehensive systematic literature search was carried out in the electronic databases including Cochrane Central Register of Controlled Trials, Web of Sciences, MEDLINE (PubMed), Scopus, EMBASE, Google Scholar, Science Direct, ProQuest, and Clininaltrial.gov, as well as Magiran, Scientific Information Database (SID), Irandoc, and Iranmedex for Persian literature up to July 2022 using Medical Subject Headings terms and Boolean operators. The keywords alone or in combination with the search for articles including BC and omega‐3 FAs were used (Table S1). The search for published or ahead‐of‐print articles was done regardless of time, place, and language limitations. Furthermore, reference lists of included articles were reviewed to identify eligible studies that had not been attained by the literature search. The conference papers were also reviewed.

In order to manage the findings of the search by the mentioned strategies, Endnote X8 software (Thomas Reuters) was employed.

Two independent review team members (AMH and ShO) independently assessed the eligibility of publications on the efficacy of omega‐3 PUFAs in BC patients by reviewing the title and abstract of identified studies. During the screening of the articles, the disagreements were resolved through discussion with a third reviewer (AFKh). This process continued for the screening of full texts.

### 2.4 Data extraction

The three reviewers (AFKh, AMH, and ShO) extracted the data from all eligible studies. They transferred the necessary information into an electronic form designed for this review consisting of the following sections: authors name and the year of publication, country of study, study design, objectives, evaluated outcomes, the number of participants, intervention and control groups, follow‐up period, study results and possible side effects (Tables 1 and 2).

**TABLE 1:** The characteristics of the included studies investigating the effect of n‐3 fatty acids during treatment on the health outcomes of breast cancer patients.

ID	Authors (years)	Study design	Country	Study objectives	Intervention group (IG), Control group (CG), (*n* = number), duration	Participants	Results	Adverse events
1	Bougnoux et al. (2009)	Single‐arm phase II trial	France	The efficacy and safety of adding DHA to a reactive oxygen species (ROS)‐generating chemotherapy regimen, which is an anthracycline‐based regimen (FEC)	IG = DHASCO (DHA Single Cell Oil) capsules (200 mg) at the time of breakfast, lunch, and dinner, three capsules were taken, in general, and nine capsules (1.8 g/day in total) were consumed daily (*n* = 25) Duration: before to 5 months after chemotherapy	Patients with visceral metastases without amenable to hormonal therapy and earlier chemotherapy for metastases with a median age (range): 58 (32–71) years	The objective response rate as primary outcome was 44% with a mean follow‐up time of 31 months (range 2–96 months). Secondary outcomes Median TTP (time to progression) High‐plasma DHA group: 8.7 months, *p* = .02 Low‐DHA plasma group: 3.5 months Median OS (overall survival) High‐plasma DHA group: 34 months, *p* = .007 Low‐plasma DHA group: 18 months	Without adverse events
2	Bjørklund (2015)	Single arm	Rana, Norway	Test the possibility of a synergistic effect of various categories of nutritional supplements	IG = patients received a cocktail consisting of vitamin C, vitamin E, beta‐carotene, Se, various other vitamins, and essential trace elements, essential fatty acids (1.2 g gamma linolenic acid/day and 3.5 g omega‐3 PUFAs/day), and coenzyme. (*n* = 32) Duration: For 5 years	Patients with breast cancer and classified as high risk who were starting standard treatment, age range: 32–81 years	Due to dying the main investigator, the study outcome was reported briefly after 18 months: None of the patients died during the study period (the expected number was 4); None of the patients showed signs of further distant metastases; The quality of life was improved	Not reported
3	DelaRosa‐Oliva et al. (2017)	RCT	Mexico	Determine the benefits of supplementation with EPA and DHA polyunsaturated fatty acids in terms of chemotoxicity and inflammatory status	IG = 2.4 g/day of PUFA Ω‐3 (EPA 1.6 g and DHA 0.8 g) (*n* = 22) CG = Placebo (*n* = 22) Duration: For 6 months	Participants with LABC (stages IIA to IIIA) Intervention during NeoCT, mean age in IG = 51.6, and mean age in CG = 49.1 years	After supplementation for 6 months, IG had significant reductions (time 0 vs. 6, respectively) in total leukocytes (from 6.3 to 5.1 × 10^3^/mL, *p* = .002), lymphocytes (from 33.5% to 25.2%, *p* = .002), leptin (from 60.2 to 36.1 pg/mL, *p* = .04), and adiponectin (from 36.8 to 43.3 mg/mL, *p* = .05). In contrast, the placebo group had significant increases in the number of leukocytes (from 6.8 to 10.0 × 10^3^/mL, *p* = .04), monocytes (from 6.7% to 10.1%, *p* = .03), and an increase in leptin (from 22.7 to 46.3 pg/mL, *p* = .04)	Not reported
4	Paixão et al. (2017)	RCT	Brazil	To evaluate the effects of EPA‐ and DHA‐enriched fish oil on nutritional and immunological parameters of treatment naïve breast cancer patients without metastasis	IG = 2 g/ day of FO concentrate containing 1.8 g of n‐3 fatty acids (*n* = 23) CG = 2 g/ day of mineral oil (*n* = 22) Duration: For 30 days	Patients with mammographic image classification 4C or higher according to Breast Imaging‐Reporting and Data System, surgery was the primary treatment option. The intervention had been done before surgery, with mean (SD) age IG: 48.6 (±9.0) years and mean (SD) age CG: 53.4 ± 7.5 years	Nutritional status (primary outcome) Fat mass (kg) Median IG (initial: 26.3, final: 26.8) *p* = .029 Median CG (initial: 26.5, final: 24.3) *p* = .977 *p* = .101 (between groups) Weight, body mass index (BMI), lean body mass, and standardized phase angle had no significant within and between groups differences (*p* ≥ .05) Blood fatty acids profile (secondary outcomes) EPA Median IG (initial: 0.4, final: 1.5) *p* = .004 Median CG (initial: 0.3, final: 0.5) *p* = .293 *p* = .034 (between groups) DHA Median IG (initial: 2.5, final: 4.6) *p* = .007 Median CG (initial: 3.1, final: 3.8) *p* = .904 *p* = .000 (between groups) Total n‐3 Median IG (initial: 3.3, final: 6.5) *p* = .004 Median CG (initial: 3.7, final: 4.1) *p* = .952 *p* = .005 (between groups) n‐6:n‐3 ratio Median IG (initial: 7.7, final: 3.8) *p* = .002 Median CG (initial: 7.0, final: 6.8) *p* = .904 *p* = .012 (between groups) Biochemical parameters (secondary outcomes) No significant change was observed. Immunological response (secondary outcomes) HsCRP (high sensitivity C‐reactive protein) Median IG (initial: 0.1, final: 0.3) *p* = .510 Median CG (initial: 0.1, final: 0.2) *p* = .0240 Variation after treatment Δ% (IG: 5.9, CG: 17.2, *p* = .059) No significant changes in serum IL‐6, TNF‐α, and IL‐1β cytokines were shown. CD4^**+**^ T lymphocytes No significant change in IG group Median CG (initial: 57.2, final: 52.7, *p* = .042)	No between‐ group differences were indicated for the presence of symptoms (*p* = .616)
5	Shen et al. (2018)	RCT	United States of America	To evaluate the effect of Omega‐3 fatty acids on aromatase inhibitor‐related arthralgia in obese breast cancer patients stages I–III	IG: received O3‐FA (3.3 g) per day; 560 mg eicosapentaenoic acid plus docosahexaenoic acid in a 40:20 ratio per day CG: received placebo (soybean–corn oil blend) BMI < 30 (kg/m^2^) *n* = 149 BMI **≥**30 (kg/m^2^) *n* = 110 Duration: For 24 weeks	Participants were postmenopausal women with stages I–III breast cancer, median age: 59 years	BPI worst pain BMI ≥ 30 kg/m^2^ Mean (SD) for week 24 (IG vs. CG): 4.36 (2.76) vs. 5.70 (2.61), *p* = .02 BPI average pain BMI ≥ 30 kg/m^2^ Mean (SD) for week 24 (IG vs. CG): 3.36 (2.22) vs. 4.65 (2.29), *p* = .002 Interaction between BMI (< 30 vs. ≥ 30 kg/m^2^) and treatment group (IG vs. CG) for week 24: *p* = .005 BPI pain Interference BMI ≥ 30 kg/m^2^ Mean (SD) for week 24 (IG vs. CG): 2.15 (2.02) vs. 3.49 (2.52), *p* = .009 Interaction between BMI (< 30 vs. ≥ 30 kg/m^2^) and treatment group (IG vs. CG) for week 24: *p* = .01 Global rating of change in joint stiffness BMI ≥ 30 kg/m^2^ Mean (SD) for week 12 (IG vs. CG): 0.93 (1.29) vs. 0.30 (1.15), *p* = .02 BMI < 30 kg/m^2^ No significant change was observed in above BPI scores for this group Lipid profile HDL (mg/dL) BMI < 30 kg/m^2^ Mean (SD) for week 24 (IG vs. CG): 69.5 (20.7) vs. 58.6 (19.1), *p* = .002 Interaction between BMI (<30 vs. ≥30 kg/m^2^) and treatment group (IG vs. CG) for week 24: *p* = .003 BMI > 30 kg/m^2^ No significant change TG (mg/dL) BMI < 30 kg/m^2^ Mean (SD) for week 12 (IG Vs. CG): 101.4 (56.0) vs. 145.3 (107.7), *p* = .02 BMI ≥ 30 kg/m^2^ Mean (SD) for week 12 (IG vs. CG): 131.2 (63.8) vs. 157.3 (82.4), *p* = .02 Interaction between BMI (<30 vs. ≥30 kg/m^2^) and treatment group (IG vs. CG) for week 24: *p* = .01 Cholesterol (mg/dL) No significant changes were observed at all	Not reported
6	Darwito et al. (2019)	RCT	Indonesia	Effects of omega‐3 supplementation on Ki‐67 and VEGF expression levels and clinical outcomes of locally advanced breast cancer patients treated with neoadjuvant CAF (cyclophosphamide, adriamycin, and fluorouracil) chemotherapy	IG: Omega‐3 fatty acids (1 g/day) *n* = 24 CG: Placebo *n* = 24 Duration: for 51 days	Participants with a confirmed diagnosis of breast cancer, advanced stage IIIB, and ductal invasive breast cancer were studied. The intervention was concurrent with three cycles of neoadjuvant chemotherapy, age range: 25–60 years	Expression levels of Ki‐67 after NeoCT: Decreased significantly in both groups. Mean (SD) for Ki‐67 (IG vs. CG): 42.4(4) vs. 39.2(5.3), *p* = .032 Expression levels of VEGF after NeoCT: Decreased significantly in both groups. Mean (SD) for VEGF (IG vs. CG): 32.7 (5.2) vs. 29.5(5.4), *p* = .041 PFS (progression‐free survival) Mean (SD) for PFS (IG vs. CG): 28.5 (3.3) vs. 23.7 (3.6) weeks, *p* = .044, HR = 0.44 Ki‐67 and VEGF expression levels at baseline were significant predictors for shorter PFS (HR 1.44, 95% CI: 1.05–2.21, *p* = .039); HR 1.33 95% CI: 1.045–2.115, *p* = .043 OS Mean (SD) for OS (IG vs. CG): 30.9 (3.7) vs. 25.9 (3.6) weeks, *p* = .048, HR = 0.411 Expression levels of Ki‐67 and VEGF were significant predictive markers for shorter overall survival (HR = 1.46. 95% CI: 1.052–2.120, *p* = .042) and HR = 1.32, CI 95% = 1.091–2.083	Diarrhea (temporal in 3 patients)
7	de la Rosa Oliva et al. (2019)	RCT	Mexico	To evaluate the effects of PUFA omega‐3 on the toxicity, side effects, body composition, cardiometabolic profile, and quality of life in women with LABC (locally advanced breast cancer) standard neoadjuvant chemotherapy	IG: received four capsules contain PUFA omega‐3 (daily dosed 2.4 g in a 2:1 ratio of DHA/EPA) *n* = 27 CG: placebo (sunflower) *n* = 26 Duration 6 months	Participants with a diagnosis of LABC confirmed by histopathology, in a clinical‐stage IIA–III B. The intervention was concurrent with the NeoCT, age range: 18–80 years	Body composition Skeletal muscle mass index (kg/m^2^) Mean (SE) for basal (IG, CG): (5.83 [0.12], 5.9 [0.15]) Mean (SE) for 6 months (IG, CG): (6.0 [0.14], 6.1 [0.15]) (basal vs. 6 months): *p* = .02 Fat mass MG (kg) Mean (SE) for basal (IG, CG): 28.5 (1.3), 28.3 (1.5) Mean (SE) for 6 months (IG, CG): (27 (1.2), 28.4 (1.4)) (basal vs. 6 months): *p* = .02 Percent body fat (%) Mean (SE) for basal (IG, CG): (41.5 [1], 40.7 [1.2]) Mean (SE) for 6 months (IG, CG): (40.6 [0.9], 40.6 [0.9]) (basal vs. 6 months): *p* = .02 Weight and BMI: no significant differences were observed following intervention Cardiometabolic profile TG “Significant increase in the concentration of TGs at three and six months (*p* = .0001) was found in both groups.” Cholesterol “Cholesterolemia was significantly higher at three and six months (*p* = .04), but no differences were found when comparing the effect of the supplementation with PUFA Ω‐3 vs. placebo.” HDL “HDL presented a significant reduction in both groups (*p* = .0001); this effect was not significant when it was compared between the two groups.” Serum albumin showed a statistically significant reduction (*p* = .0001), however, this effect was not significant in relation to PUFA Ω‐3 or placebo groups. Liver assessment Albumin “Serum albumin showed a statistically significant reduction (*p* = .0001), however, this effect was not significant in relation to PUFA Ω‐3 or placebo groups.” ALT(alanine aminotransferase) “The two groups show an increase considered to be still normal, which was significant at three and six months (*p* = .002)”	Without adverse events related to the use of the PUFA Ω‐3
8	Kahlenberg et al. (2020)	Prospective, comparative, two‐arm, interventional study (brief report)	United States of America	To assess the impact of omega‐3 fatty acid supplementation on the incidence of surgical site healing complications and surgical site infections surgical site infections	IG: omega‐3 FFAs (free fatty acids), 2700 mg, oral, twice daily + letrozole CG: letrozole 2.5 mg by oral/daily n = Patients were randomized to one of two arms of 100 patients. Duration: 21–30 months	Interventions were before undergoing their surgery, age range: 49–88 years	“The incidence of healing complications and infections was 8% in the omega‐3 group (n = 25) compared to 33% in the control group (n = 6). This however was not statistically significant (*p* = .159)”	Not reported

*Note*: *p*‐Value < .05 considered as statistically significant.

Abbreviations: BMI, body mass index; BPI, Brief Pain Intensity; CRP, C‐reactive protein; DHA, docosahexaenoic acid; EPA, eicosapentaenoic; HDL, high‐density lipoprotein; HR, Hazard Ratio; NeoCT, neoadjuvant chemotherapy; OS, overall survival; PUFA Ω‐3, polyunsaturated fatty acids; RCT, randomized control trial; SD, standard deviation; SE, standard error; TG, triglyceride; VEGF, Vascular endothelial growth factor; y, year.

**TABLE 2:** The characteristics of the included studies investigating the effect of n‐3 fatty acids following primary medical treatment on the health outcomes of breast cancer patients.

ID	Authors (years)	Study design	Country	Study objectives	Intervention group (IG) control group (CG; *n* = number) duration	Participants	Results	Adverse events
1	Martínez et al. (2019)	Single‐arm, clinical trial	Madrid, Spain	“To evaluate whether the olive‐derived polyphenol hydroxytyrosol combined with omega‐3 fatty acids and curcumin would reduce CRP and musculoskeletal symptoms in The American Joint Committee on Cancer (AJCC)Stage 0‐III breast cancer patients receiving adjuvant hormonal therapies”	IG: participants received three capsules per day (two in the morning during breakfast and one in the evening during dinner). Each active capsule contained 460 mg of fish oil (EPA and DHA), 125 mg of Hytolive® powder (12.5 mg of natural hydroxytyrosol), and 50 mg extract of curcumin (47.5 mg curcuminoids) (n:45) Duration: For 1 month	Participants were postmenopausal women with histopathological diagnoses of AJCC stages 0–IIIA. Intervention during hormone therapy and more than 12 months since primary breast cancer surgery, at least 6 months since last chemotherapy, median age (range): 57.3 (40–81) years	Change in CRP levels (primary outcome) CRP > 9.75 Mean change days 0–30 (−12.0 ± 16.4 (% reduction 49.4), *p* = .019) Mean change days 0–60 (−13.0 ± 17.3 (% reduction 50.7), *p* = .032) 5.85 < CRP ≤ 9.75 Mean change days 0–30 (−2.2 ± 3.0, (% reduction 26.2), *P* = .038) Mean change days 0–60 (−2 ± 5.1, (% reduction 25.2), *p* = .278) CRP ≤ 5.85 Mean change days 0–30 (−0.2 + 2.9, (% reduction 3.1), *p* = .733) Mean change days 0‐60 (0.6 ± 4.3 (% reduction‐12.2), *p* = .560) BPI (secondary outcome) pain at its worst in the last 24 h Mean change days 0–30 (− 1.6 ± 3 (% reduction 21.5), *p* = .011) pain at its least in the last 24 h Mean change days 0–30 (−0.9 ± 2.1 [% reduction 20.3], *p* = .045) pain on average Mean change days 0–30 (−0.8 ± 2.4 [% reduction 17.2], *p* = .119) how much pain do you have right now Mean days 0–30 (−1.5 ± 2.3 [% reduction 45.9], *p* = .004) Pain severity Mean day 0–30(−1.2 ± 2.2, [% reduction 26.6], *p* = .008) BPI Total Score Mean day 0–30 (−6.5 ± 17.1, [% reduction 9.1], *p* = .068) Lipid profile (secondary outcome) TG: A 7% decrease (*p* = .011) HDL: did not differ from basal values LDL (low‐density lipoprotein): A non‐significant increase	In general, the EPA/DHA/hydroxytyrosol/curcumin was associated with few adverse events. Constipation and abnormal or a fish taste were the most common. No clinically relevant adverse events were reported
2	Kleckner et al. (2021)	RCT, phase II	United States of America	To investigate whether nutritional status was associated with the uptake of the fatty acids or the associations between serum omega‐3 concentrations	IG_1_: 6 g fish oil (daily) *n* = 30 CG: 6 g soybean oil (daily) *n* = 30 IG_2_: 3 g of each (daily) *n* = 23 Duration: For 6 weeks	Participants with stages 0–III breast cancer. Interventions were 4–36 months post‐treatment (surgery, radiation, and/or chemotherapy) with moderate–severe fatigue mean age: 61.2 years	Fatigue (primary outcome) Fatigue significantly improved over time for participants in all groups (*p* < .001) Among the fish oil group, greater changes in omega‐3 fatty acids in the blood were associated with greater improvements in fatigue over the course of the intervention. Physical fatigue (*β* ± SE = 4.53 ± 1.98, *p* = .022) Vigor (*β* ± SE = 4.85 ± 2.04, *p* = .018) Change in fatty acids (secondary outcome) Total omega‐3 fatty acids (mM) Mean (SE) CG**: −**0.01 (0.09) Mean (SE) IG_2_: 0.38 (0.19) *p* < .001 Mean (SE) IG_1_: 0.59 (0.33) DHA (μM) Mean (SE) CG: −8.30 (44.82) Mean (SE) IG_2_: 164.83 (80.48) *p* < .001 Mean (SE) IG_1_: 207.13 (101.79) EPA (μM) Mean (SE) CG: −5.04(31.44) Mean (SE) IG_2_: 187.78(107.86) *p* < .001 Mean (SE) IG_1_: 358.00 (223.02) Total omega‐6 fatty acids (mM) Mean (SE) CG: 0.05 (0.83) Mean (SE) IG_2_: −0.11 (0.73) *p* = .008 Mean (SE)IG_1_: −0.11 (0.73) Arachidonic acid(μM) Mean (SE) CG: −31.78 (180.74) Mean (SE) IG_2_: −105.48(169.55) *p* = .001 Mean (SE)IG_1_: −208.20 (172.49) Omega‐6:omega‐3 ratio Mean (SE) CG: 0.57 (2.94) Mean (SE) IG_2_: 9.13 (3.80) *p* < .001 Mean (SE)IG_1_: 11.19 (6.94)	Not reported
3	Xu et al. (2022)	RCT	United States of America	Investigating the reduction of psycho‐neurological symptoms with a personalized meal plan with dietary omega‐3LC	Participants received wild‐caught Alaskan salmon IG_1_: high omega‐3LC (12 ounces/week, 2040 mg omega‐3LC) *n* = 24 IG_2_: low omega‐3LC (6 ounces/week, 1020 mg) *n* = 15 Duration: Phase I: 3 weeks Phase II: 6 weeks	Participants were 6–24 months post‐treatment for early‐stage BC (stage I to IIIA), ages: 30–75 years	Psycho‐neurological symptoms High omega‐3LC dietary group had a significant decrease in perceived stress (*p* < .05), depression (*p* < .001), sleep (*p* < .001), pain (BPI total pain, *p* < .01), and fatigue (*p* < .01) following intervention. Low omega‐3LC group had trends of symptom improvement; however, the difference did not reach statistical significance. Cohen's *d* (the effect size of the mean) for the pre–post changes (IG_1_ vs. IG_2_): Mean (±SD) perceived stress scale: –2.58 (±6.34), *p* < .05, Cohen's *d* (95% CI): 0.41(0.14–0.67) Mean (±SD) general sleep disturbance scale: –7.61 (± 17.59), *p* < .05 Cohen's *d* (95% CI): 0.43 (−0.10 = 0.86) Mean (±SD) CES‐D (Center for Epidemiological Studies Depression scale): –3.82 (±6.90), *p* < .01 Cohen's *d* (95% CI): 0.55 (0.32–0.86) Mean (±SD) BPI total: −4.58 (±19.24), *p* = .09 Cohen's *d* (95% CI): 0.24 (−0.08‐0.66) Mean (±SD) BFI (Brief Fatigue Inventory) total: –9.15 (±21.80), *p* < .05 Cohen's *d* (95% CI): 0.42 (0.08–0.88) Nutrition consumption in both groups Omega 6:omega 3 ratios: *p* < .001 Vitamin D: *p* < .001 Vitamin E: *p* < .01	No intervention‐related adverse events were reported

*Note*: *p*‐Value < .05 was considered as statistically significant.

Abbreviations: BPI, Brief Pain Intensity; DHA, docosahexaenoic acid; EPA, eicosapentaenoic; RCT, randomized control trial; SD, standard deviation; SE, standard error; y, year.

### 2.5 Assessment of methodological quality

The risk of bias and quality of the included articles were assessed through the guidance of the Cochrane Collaboration Handbook by two members of the review (AFKh & AMH). The following items were assessed: random sequence generation (selection bias), allocation concealment (selection bias), blinding of participants and personnel (performance bias), blinding of outcome assessment (detection bias), incomplete outcome data (attrition bias), and selective reporting (reporting bias). Disagreements were resolved through discussion with a third party (ShO). The bias of the studies was demonstrated using Review Manager 5.3 (RevMan; The Cochrane Collaboration, Oxford, UK) software.

### 2.6 Data synthesis

Data were synthesized through qualitative approaches and reported as a systematic review because there were inadequate data for data pooling. Three articles were single‐arm interventional studies. The remaining eight studies had not enough similar outcomes to meta‐analysis.

## 3 RESULTS

### 3.1 Literature search and selected articles

**FIGURE 1:**
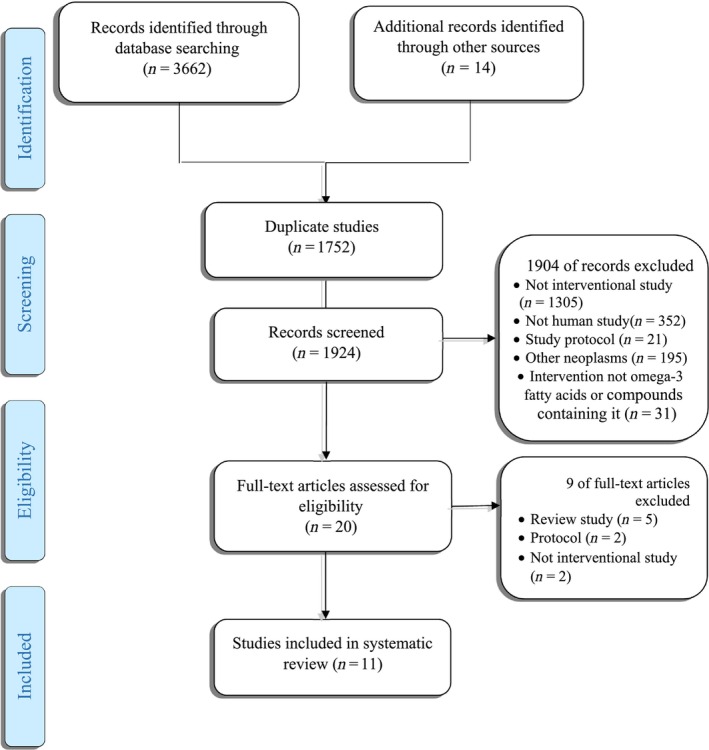
Diagram for the search and selection process of the articles considered in this review.

Details of the search process and article selection, as well as the reasons for article exclusion, are presented in Figure 1. Of 3676 identified articles in the search of various databases, 1752 articles were removed from the study as duplications, and the rest of the trials were screened to evaluate the eligibility criteria. Among the reviewed 1924 articles, 1904 was excluded due to not being an interventional study (*n* = 1305), not being a human study (*n* = 352), being a study protocol (*n* = 21), investigating other neoplasms (*n* = 195), and intervention by supplements other than omega‐3 FAs or compounds containing them (*n* = 31). Although 20 studies were selected for systematic review by surveying the title and abstract of the articles, after reviewing the full texts, 11 articles were eligible and finally included in this systematic review.

### 3.2 Description of the studies

The selected trials in this systematic review have been published from 2009 to 2022. Of the 11 articles reviewed, 10 articles had full English text (Bjørklund, 2015; Bougnoux et al., 2009; Darwito et al., 2019; de la Rosa Oliva et al., 2019; Kahlenberg et al., 2020; Kleckner et al., 2021; Martínez et al., 2019; Paixão et al., 2017; Shen et al., 2018; Xu et al., 2022) and one article had English abstract (DelaRosa‐Oliva et al., 2017). Among included articles, eight studies were RCTs and three of them were single‐arm uncontrolled before–after trials (Bjørklund, 2015; Bougnoux et al., 2009; Martínez et al., 2019). In addition, four articles were conducted in the United States (Kahlenberg et al., 2020; Kleckner et al., 2021; Shen et al., 2018; Xu et al., 2022), two articles in Mexico (de la Rosa Oliva et al., 2019; DelaRosa‐Oliva et al., 2017), and one article in Norway (Bjørklund, 2015), Spain (Martínez et al., 2019), Indonesia (Darwito et al., 2019), Brazil (Paixão et al., 2017), and France (Bougnoux et al., 2009). The total number of participants in this systematic review was 678. The stage of BC was 0‐III. Omega‐3 FAs were supplemented in combination with other nutrients only in two studies (Bjørklund, 2015; Martínez et al., 2019) and used alone in the rest of the studies (Bougnoux et al., 2009; Darwito et al., 2019; de la Rosa Oliva et al., 2019; DelaRosa‐Oliva et al., 2017; Kahlenberg et al., 2020; Kleckner et al., 2021; Paixão et al., 2017; Shen et al., 2018; Xu et al., 2022). In one study, wild‐caught Alaskan salmon consumption in two different amounts per week was the source of omega‐3 FAs (Xu et al., 2022). In eight studies, omega‐3 FAs were supplemented during BC medical treatment, and in three studies, omega‐3 FAs supplementation was done during treatment follow‐up (Kleckner et al., 2021; Martínez et al., 2019; Xu et al., 2022). All patients were supplemented orally and the pharmaceutical form was oral capsules in most articles. The dosage and duration of the intervention varied in most studies. Daily omega‐3 FAs supplementation doses ranged from 300 to 6000 mg. Additionally, the intervention duration ranged from 21 to 30 days to 5 years. Detailed characteristics of all articles are listed in Tables 1 and 2 & Table S2.

### 3.3 Risk of bias in the included studies

**FIGURE 2:**
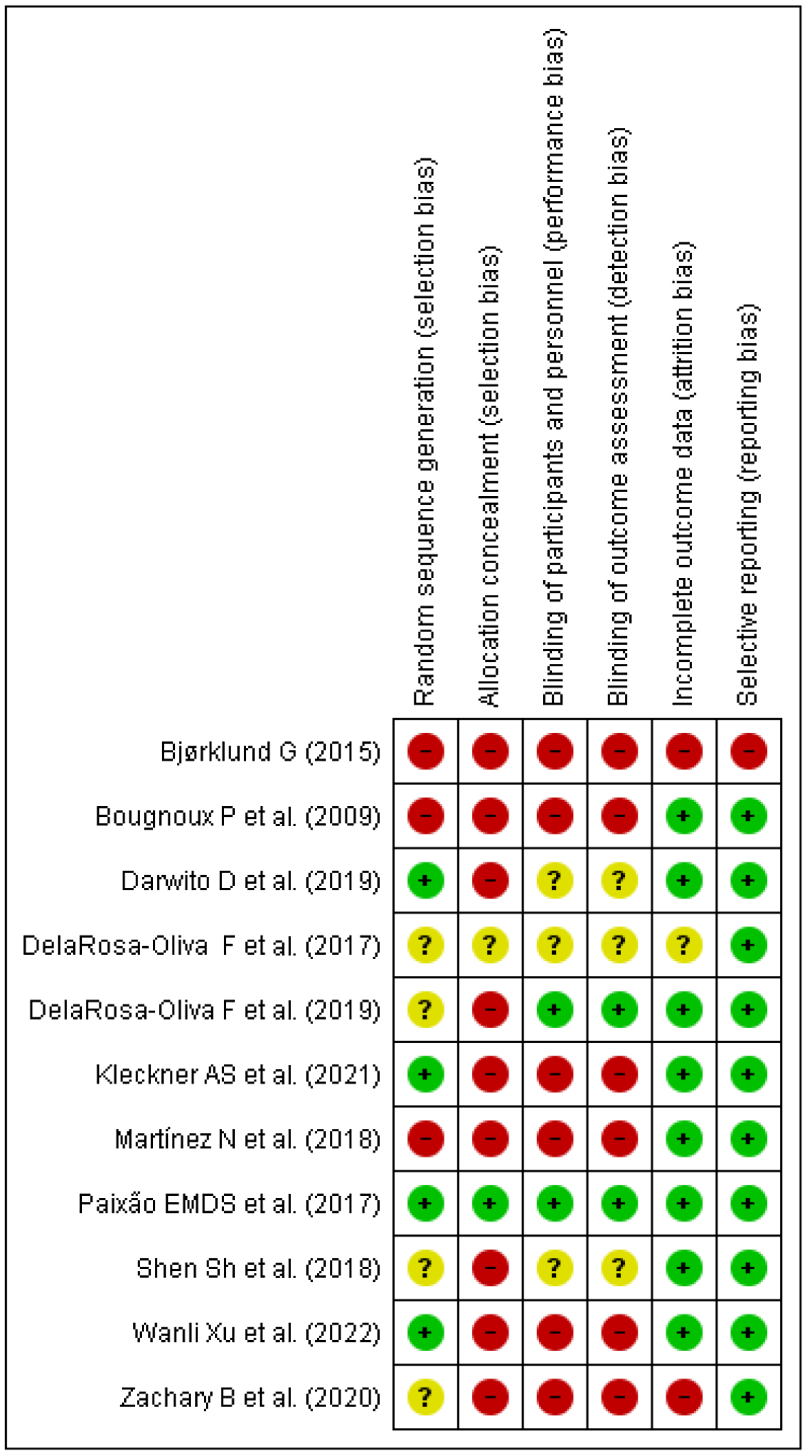
Risk of bias summary: review authors' judgments about each risk of bias item for each included study.

**FIGURE 3:**
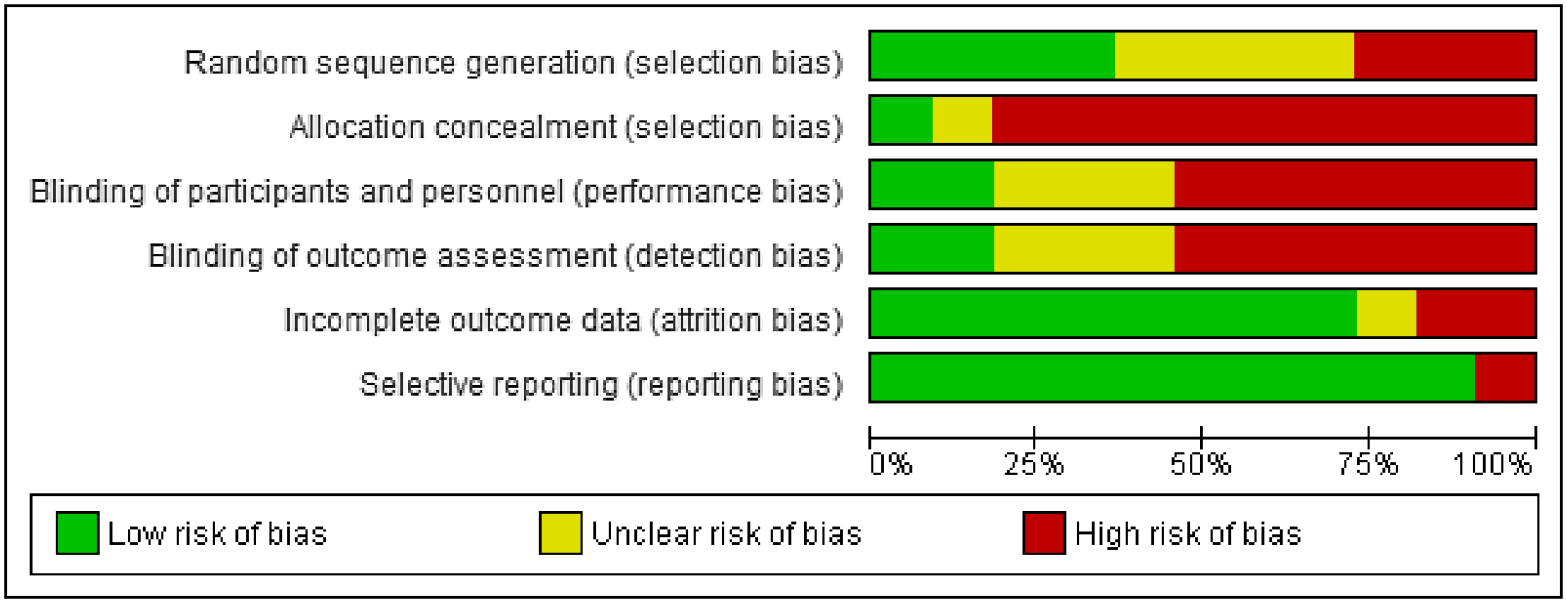
Risk of bias graph: review authors' judgments about each risk of bias item presented as percentages across all included studies.

The methodological quality based on the researchers' decision on every risk of bias for each included article is illustrated in Figures 2 and 3. The majority of the included studies were not of high quality.

### 3.4 Effectiveness of the interventions

Bjørklund (2015) investigated the possibility of a synergistic effect of various categories of nutritional supplements including essential fatty acids (after 18 months). They reported that none of the patients had died during the study period. None of them showed signs of further distant metastases. The quality of life was improved (no weight loss and reduced use of painkillers). Subsequently, there were some cases of death, but not more than 5 or 6 (while 16 deaths were expected from historical controls) after 4.5 years. Darwito et al. (2019) in their double‐blind RCT indicated that post‐intervention progression‐free survival (PFS; *p* = .044) and OS (*p* = .048) were significantly longer in the intervention than the placebo group. The expression levels of proliferation indices including Ki‐67 and vascular endothelial growth factor (VEGF) significantly decreased after the intervention. Bougnoux et al. (2009) in their trial reported 22 months of median OS which reached 34 months in patient sub‐population with the highest plasma DHA incorporation. Martínez et al. (2019) also indicated that CRP (*p* = .014) and pain at its worst in the last 24 h (*p* = .011) were reduced on day 30 after the intervention. Regarding lipid profile, triglyceride (TG) was reduced by 7% (*p* = .011). In the study by Shen et al. (2018), brief pain inventory (BPI) worst pain (*p* = .02), BPI average pain (*p* = .002), and BPI pain interference (*p* = .009) significantly reduced in week 24 in patients who had body mass index (BMI) ≥ 30 kg/m^2^ compared to placebo. The global rating of change in joint stiffness also significantly decreased in week 12 (*p* = .02). High‐density lipoprotein cholesterol (HDL‐c) in week 24 significantly increased in supplemented women with BMI < 30 kg/m^2^ in comparison with placebo (*p* = .002). TG in week 12, in patients with BMI < 30 and BMI ≥ 30 kg/m^2^, significantly decreased compared to placebo (*p* = .02). In double‐blind RCTs by Paixão et al. (2017) and Kleckner et al. (2021), total omega‐3 (*p* = .005), EPA (.034), and DHA (*p* < .001) significantly increased, and omega‐6:omega‐3 ratio (*p* = .012) decreased in the intervention group compared to the placebo counterparts.

DelaRosa‐Oliva et al. (2017) conducted a double‐blind RCT. After 6 months, patients who were supplemented with PUFAs showed a significant reduction in total leukocytes (from 6.3 to 5.1 × 10^3^/mL, *p* = .002), lymphocytes (from 33.5 to 25.2%, *p* = .002), leptin (from 60.2 to 36.1 pg/mL, *p* = .04), and adiponectin (from 36.8 to 43.3 mg/mL, *p* = .05). de la Rosa Oliva et al. (2019) in their double‐blind RCT also reported a significant decrement in body fat at 3 and 6 months in the omega‐3 PUFAs‐supplemented group (*p* = .02). However, serum lipid profile and serum albumin were not different in the intervention compared to the placebo group. Kahlenberg et al. (2020) in their RCT showed a trend toward reduced surgical site healing complications (SSHCs) and surgical site infections (SSIs) in patients who received omega‐3 FAs supplementation. The incidence of SSHCs and SSIs was 8% in the omega‐3 group compared to 33% in the control group. However, this difference was not statistically significant (*p* = .159). Xu et al. (2022) in their RCT study reported that BC survivors receiving a high omega‐3LC dietary group (*n* = 20) had a significant decrease in perceived stress (*p* < .05), sleep disturbance (*p* < .001), depression (*p* < .001), pain (BPI total pain, *p* < .01), and fatigue (*p* < .01) compared to the low omega‐3LC dietary group over the course of intervention (Tables 1 and 2).

## 4 DISCUSSION

This study aimed to review the existing studies on the effect of omega‐3 FAs on health outcomes in BC patients under therapeutic regimens. Overall, the results of the present study indicated the positive effects of these FAs on treatment outcomes. However, some studies reported no significant changes or side effects in studied parameters after taking omega‐3 FAs in BC patients.

Omega‐3 FAs are essential fatty acids with diverse potential biological effects. In recent decades, the benefits of these fatty acids on health outcomes have also been further studied. Due to the presence of two or more unsaturated bonds, these fatty acids possess an unstable structure, enabling them to keep the suitable membrane fluidity and related signaling. However, high temperature, oxidation, and hydrogenation will result in free radicals formation from omega‐3 FAs, which are harmful mediators in the human body (Yashodhara et al., 2009). Hence, omega‐3 FAs supplementation and consumption must be done very carefully.

In the present study, we evaluated the effect of omega‐3 FAs on different parameters in BC patients under different therapeutic regimens. Regarding the survival and patients' quality of life, gamma‐linolenic acid and omega‐3 FAs supplementation (1.800 mg DHA/day) for 5 months after chemotherapy resulted in increased survival, improved quality of life, and fewer deaths compared to the placebo group (Bougnoux et al., 2009). Moreover, Shen et al. (2018) reported a decreased pain degree in obese BC patients (BMI > 30 kg/m^2^) suffering from aromatase inhibitor‐related arthralgia after omega‐3 FAs supplementation (3.3 g/day) for 24 weeks. Darwito et al. (2019) also reported a longer PFS and OS in omega‐3‐supplemented patients (1 g/day) compared to the placebo group after 48 weeks in IIIB stage BC patients receiving neoadjuvant chemotherapy. Moreover, Bougnoux et al. (2009) reported the beneficial effects of DHA addition (1.800 mg DHA/day) to ROS‐generating chemotherapy regimen on objective response rate, TTP, and OS in BC patients. Omega‐3 FAs supplementation (12 ounces/week, 2040 mg omega‐3LC) also significantly decreased the perceived stress, sleep disturbance, depression, pain, and fatigue in BC survivors (Xu et al., 2022). Combination of olive‐derived polyphenol hydroxytyrosol, omega‐3 FAs, and curcumin (each active capsule contained 460 mg of fish oil (EPA and DHA), 125 mg of Hytolive® powder (12.5 mg of natural hydroxytyrosol), and 50 mg extract of curcumin (47.5 mg curcuminoids)) for 1 month also significantly reduced the CRP and TG levels, as well as pain in BC patients receiving hormonal therapy. However, no significant changes were observed regarding the other inflammatory factors in the current study (Martínez et al., 2019). Paixão et al. (2017) in their study also reported a nonsignificant difference in basal hs‐CRP levels in BC patients receiving EPA‐ and DHA‐supplemented fish oil (2 g/day of FO concentrate containing 1.8 g of omega‐3 fatty acids) after 30 days, however, this parameter was significantly increased in the placebo group. Omega‐3 FAs supplementation also exhibited a beneficial effect on the biochemical and lipid profile of the patients. Martínez et al. (2019) reported a significant decrease in TG levels after omega‐3 FAs supplementation. Shen et al. (2018) also showed decreased TG levels in BC patients with BMI ≤ 30 kg/m^2^ and BMI ≥ 30 kg/m^2^ after omega‐3 supplementation (3.3 g/day for 24 weeks). Additionally, HDL significantly increased in BC patients with BMI ≤ 30 kg/m^2^. Paixão et al. (2017) also reported a significant increase in total omega‐3, EPA, and DHA, and a significant reduction in omega‐6:omega‐3 ratio in BC patients receiving EPA‐ and DHA‐supplemented FO (2 g/day of FO concentrate containing 1.8 g of omega‐3 fatty acids) after 30 days. Similar results were also reported by Kleckner et al. (2021) regarding the increased total serum omega‐3 FAs (EPA and DHA) following daily FO consumption (6 g/day fish oil for 6 weeks) compared to the increased levels of omega‐6 FAs (such as arachidonic acid) in soybean oil receiving (6 g/day soybeans for 6 weeks) controls. Arachidonic acid is considered the main precursor of pro‐inflammatory mediators including prostaglandins and leukotriene, leading to the development of inflammatory responses, and hence may worsen the disease severity (Innes & Calder, 2018).

However, despite a significant decrease in body fat in the omega‐3 FAs‐supplemented group in LABC patients, 2.4 g/day of PUFA omega‐3 (EPA 1.6 g and DHA 0.8 g) for 6 months, DelaRosa Oliva et al. (2019) in their study showed an increased level of TG and cholesterol in the mentioned group. This phenomenon may be due to the BC type, differences in ethnicity, chemotherapeutic regimen, or patients' nutritional condition.

Increased expression of proliferation indices, including Ki‐67 and VEGF reported by Darwito et al. (2019) after omega‐3 FAs supplementation (1 g/day for 51 days), is to some extent controversial. As reported by Bonetti et al. (1996), higher cell proliferation may be associated with improved response to chemotherapy in a short time. Omega‐3 supplementation in Darwito et al. (2019) study led to increased Ki‐67 and VEGF after 48 weeks of follow‐up, which was also associated with increased OS and PFS in the studied population. In a short time, this effect may be beneficial, however, long‐term follow‐up is needed to better evaluate the effect of this supplementation on patients' OS and PFS.

Regarding the immune system responses, omega‐3 FAs supplementation also resulted in unchanged CD4+ and CD8+ T cells in BC patients without metastasis receiving EPA‐ and DHA‐supplemented FO (2 g/day of FO concentrate containing 1.8 g of omega‐3 fatty acids for 30 days) in Paixão et al.'s (2017) study. In the placebo group, a significant reduction in CD4+ T cells was observed. These data may suggest the positive effect of omega‐3 FAs supplementation on adaptive immunity. However, to better understand the data, it would be more beneficial to evaluate the T CD4+ cell subsets, including Th17 and Treg. Moreover, DelaRosa‐Oliva et al. (2017) reported a significantly decreased total leukocytes, lymphocytes, and leptin, as well as increased adiponectin levels in LABC patients compared to the placebo group, indicating the attenuating effects of omega‐3 FAs, 2.4 g/day of PUFA ω‐3 (EPA 1.6 g and DHA 0.8 g) for 6 months on inflammatory responses.

### 4.1 The limitations of the studies and the need for further research

The included studies had some limitations, which necessitate further research regarding the effects of omega‐3 FAs supplementation in BC patients under chemotherapy regimens. Included studies were different in study design, type of BC, chemotherapy regimen, patient's BMI, disease staging, used omega‐3 FAs dose, supplementation duration, and sample sizes. Additionally, ethnic differences were another confounding factor, which may affect the results of the study. Moreover, included studies evaluated different parameters which make it difficult to compare the outcomes of the omega‐3 FAs supplementation in BC. Due to the mentioned limitation, it was impossible conducting a meta‐analysis for this systematic review.

Considering the above‐mentioned limitations, further research regarding the effect of omega‐3 FAs on health outcomes in BC patients under chemotherapy is suggested. This review focused on human articles and, as such, it is suggested that a review be made on animal subjects in this field.

## 5 CONCLUSION

This systematic review revealed that omega‐3 FAs alone or accompanied by other supplements lead to improved physical and mental symptoms, as well as some inflammatory and metabolic indices during the treatment or post‐treatment course of BC patients. Mild gastrointestinal symptoms were only reported in two studies without clinically relevant adverse events. We strongly recommend performing more high‐quality RCTs to pool the results and draw more accurate conclusions on the efficacy and safety of omega‐3 FAs supplementation on diverse health outcomes in BC patients under standard medical therapy.

## AUTHOR CONTRIBUTIONS

**Shirin Osouli‐Tabrizi:** Conceptualization (equal); data curation (equal); formal analysis (equal); writing – original draft (equal). **Amir Mehdizadeh:** Conceptualization (equal); data curation (equal); formal analysis (equal); project administration (equal); writing – original draft (equal). **Mina Naghdi:** Data curation (equal); formal analysis (equal); investigation (equal); writing – review and editing (equal). **Zohreh Sanaat:** Data curation (equal); formal analysis (equal); investigation (equal); writing – review and editing (equal). **Nafiseh Vahed:** Conceptualization (equal); data curation (equal); formal analysis (equal); investigation (equal); writing – review and editing (equal). **Azizeh Farshbafkhalili:** Conceptualization (equal); data curation (equal); investigation (equal); methodology (equal); resources (equal); validation (equal); writing – original draft (equal); writing – review and editing (equal).

## CONFLICT OF INTEREST STATEMENT

The authors declare no conflict of interest for this study.

## ETHICAL APPROVAL

No ethical conflicts are involved in this project as this work is a systematic review research of already published data.

## Supporting information


Data S1.

## Data Availability

The data that support the findings of this study are available on request from the corresponding author.
