# Supplementary material for: The effectiveness of omega‐3 fatty acids on health outcomes in women with breast cancer: A systematic review
Source: Food Sci Nutr. 2023 May 22;11(8):4355–71. doi: 10.1002/fsn3.3409 (PMC10420771; doi:10.1002/fsn3.3409)
Supplement: Supplementary file 1 — Data S1. [file FSN3-11-4355-s001.docx]

|  | **Table S1**. Search Strategy of PubMed (Breast Cancer + Omega 3) |  |
| --- | --- | --- |
| Search | Query | Items found |
| #1 | Search: "Breast Neoplasms"[Mesh] | [316,353](https://pubmed.ncbi.nlm.nih.gov/?term=%22Breast+Neoplasms%22%5BMesh%5D&sort=relevance) |
| #2 | Search: ((((((((((((((((("Breast Neoplasm*"[Title/Abstract]) OR ("Breast Tumor*"[Title/Abstract])) OR ("Breast Cancer*"[Title/Abstract])) OR ("Breast Carcinoma*"[Title/Abstract])) OR ("Breast Neoplasia*"[Title/Abstract])) OR ("Breast Malignanc*"[Title/Abstract])) OR ("Mammary Cancer*"[Title/Abstract])) OR ("Cancer of Breast"[Title/Abstract])) OR ("Cancer of the Breast"[Title/Abstract])) OR ("Neoplasia of Breast"[Title/Abstract])) OR ("Neoplasia of the Breast"[Title/Abstract])) OR ("Breast Malignant Tumor*"[Title/Abstract])) OR ("Breast Malignant Neoplasm*"[Title/Abstract])) OR ("Malignant Tumor of Breast"[Title/Abstract])) OR ("Malignant Neoplasm of Breast"[Title/Abstract])) OR ("Human Mammary Carcinoma*"[Title/Abstract])) OR ("Human Mammary Neoplasm*"[Title/Abstract])) OR ("Human Mammary Cancer*"[Title/Abstract]) | [339,425](https://pubmed.ncbi.nlm.nih.gov/?term=%28%28%28%28%28%28%28%28%28%28%28%28%28%28%28%28%28%22Breast+Neoplasm%2A%22%5BTitle%2FAbstract%5D%29+OR+%28%22Breast+Tumor%2A%22%5BTitle%2FAbstract%5D%29%29+OR+%28%22Breast+Cancer%2A%22%5BTitle%2FAbstract%5D%29%29+OR+%28%22Breast+Carcinoma%2A%22%5BTitle%2FAbstract%5D%29%29+OR+%28%22Breast+Neoplasia%2A%22%5BTitle%2FAbstract%5D%29%29+OR+%28%22Breast+Malignanc%2A%22%5BTitle%2FAbstract%5D%29%29+OR+%28%22Mammary+Cancer%2A%22%5BTitle%2FAbstract%5D%29%29+OR+%28%22Cancer+of+Breast%22%5BTitle%2FAbstract%5D%29%29+OR+%28%22Cancer+of+the+Breast%22%5BTitle%2FAbstract%5D%29%29+OR+%28%22Neoplasia+of+Breast%22%5BTitle%2FAbstract%5D%29%29+OR+%28%22Neoplasia+of+the+Breast%22%5BTitle%2FAbstract%5D%29%29+OR+%28%22Breast+Malignant+Tumor%2A%22%5BTitle%2FAbstract%5D%29%29+OR+%28%22Breast+Malignant+Neoplasm%2A%22%5BTitle%2FAbstract%5D%29%29+OR+%28%22Malignant+Tumor+of+Breast%22%5BTitle%2FAbstract%5D%29%29+OR+%28%22Malignant+Neoplasm+of+Breast%22%5BTitle%2FAbstract%5D%29%29+OR+%28%22Human+Mammary+Carcinoma%2A%22%5BTitle%2FAbstract%5D%29%29+OR+%28%22Human+Mammary+Neoplasm%2A%22%5BTitle%2FAbstract%5D%29%29+OR+%28%22Human+Mammary+Cancer%2A%22%5BTitle%2FAbstract%5D%29&ac=no&sort=relevance) |
| #3 | Search: ("Breast Neoplasms"[Mesh]) OR (((((((((((((((((("Breast Neoplasm*"[Title/Abstract]) OR ("Breast Tumor*"[Title/Abstract])) OR ("Breast Cancer*"[Title/Abstract])) OR ("Breast Carcinoma*"[Title/Abstract])) OR ("Breast Neoplasia*"[Title/Abstract])) OR ("Breast Malignanc*"[Title/Abstract])) OR ("Mammary Cancer*"[Title/Abstract])) OR ("Cancer of Breast"[Title/Abstract])) OR ("Cancer of the Breast"[Title/Abstract])) OR ("Neoplasia of Breast"[Title/Abstract])) OR ("Neoplasia of the Breast"[Title/Abstract])) OR ("Breast Malignant Tumor*"[Title/Abstract])) OR ("Breast Malignant Neoplasm*"[Title/Abstract])) OR ("Malignant Tumor of Breast"[Title/Abstract])) OR ("Malignant Neoplasm of Breast"[Title/Abstract])) OR ("Human Mammary Carcinoma*"[Title/Abstract])) OR ("Human Mammary Neoplasm*"[Title/Abstract])) OR ("Human Mammary Cancer*"[Title/Abstract])) | [415,472](https://pubmed.ncbi.nlm.nih.gov/?term=%28%22Breast+Neoplasms%22%5BMesh%5D%29+OR+%28%28%28%28%28%28%28%28%28%28%28%28%28%28%28%28%28%28%22Breast+Neoplasm%2A%22%5BTitle%2FAbstract%5D%29+OR+%28%22Breast+Tumor%2A%22%5BTitle%2FAbstract%5D%29%29+OR+%28%22Breast+Cancer%2A%22%5BTitle%2FAbstract%5D%29%29+OR+%28%22Breast+Carcinoma%2A%22%5BTitle%2FAbstract%5D%29%29+OR+%28%22Breast+Neoplasia%2A%22%5BTitle%2FAbstract%5D%29%29+OR+%28%22Breast+Malignanc%2A%22%5BTitle%2FAbstract%5D%29%29+OR+%28%22Mammary+Cancer%2A%22%5BTitle%2FAbstract%5D%29%29+OR+%28%22Cancer+of+Breast%22%5BTitle%2FAbstract%5D%29%29+OR+%28%22Cancer+of+the+Breast%22%5BTitle%2FAbstract%5D%29%29+OR+%28%22Neoplasia+of+Breast%22%5BTitle%2FAbstract%5D%29%29+OR+%28%22Neoplasia+of+the+Breast%22%5BTitle%2FAbstract%5D%29%29+OR+%28%22Breast+Malignant+Tumor%2A%22%5BTitle%2FAbstract%5D%29%29+OR+%28%22Breast+Malignant+Neoplasm%2A%22%5BTitle%2FAbstract%5D%29%29+OR+%28%22Malignant+Tumor+of+Breast%22%5BTitle%2FAbstract%5D%29%29+OR+%28%22Malignant+Neoplasm+of+Breast%22%5BTitle%2FAbstract%5D%29%29+OR+%28%22Human+Mammary+Carcinoma%2A%22%5BTitle%2FAbstract%5D%29%29+OR+%28%22Human+Mammary+Neoplasm%2A%22%5BTitle%2FAbstract%5D%29%29+OR+%28%22Human+Mammary+Cancer%2A%22%5BTitle%2FAbstract%5D%29%29&ac=no&sort=relevance) |
| #4 | Search: ("Fatty Acids, Omega-3"[Mesh]) OR "Fish Oils"[Mesh] | [32,265](https://pubmed.ncbi.nlm.nih.gov/?sort=date&term=%28%22Fatty+Acids%2C+Omega-3%22%5BMesh%5D%29+OR+%22Fish+Oils%22%5BMesh%5D) |
| #5 | Search: (((((((((((((((Omega-3[Title/Abstract]) OR (Omega 3[Title/Abstract])) OR (Omega3[Title/Abstract])) OR (n-3 Oil*[Title/Abstract])) OR (n 3 Oil*[Title/Abstract])) OR (n3 Oil*[Title/Abstract])) OR (n-3 PUFA[Title/Abstract])) OR (n 3 PUFA[Title/Abstract])) OR (n3 PUFA[Title/Abstract])) OR ("n-3 Fatty Acid*"[Title/Abstract])) OR ("n 3 Fatty Acid*"[Title/Abstract])) OR ("n3 Fatty Acid*"[Title/Abstract])) OR ("n-3 Polyunsaturated Fatty Acid*"[Title/Abstract])) OR ("n 3 Polyunsaturated Fatty Acid*"[Title/Abstract])) OR ("n3 Polyunsaturated Fatty Acid*"[Title/Abstract])) OR (("Fish Oil*"[Title/Abstract]) OR ("Fish Liver Oil*"[Title/Abstract])) | [34,397](https://pubmed.ncbi.nlm.nih.gov/?term=%28%28%28%28%28%28%28%28%28%28%28%28%28%28%28Omega-3%5BTitle%2FAbstract%5D%29+OR+%28Omega+3%5BTitle%2FAbstract%5D%29%29+OR+%28Omega3%5BTitle%2FAbstract%5D%29%29+OR+%28n-3+Oil%2A%5BTitle%2FAbstract%5D%29%29+OR+%28n+3+Oil%2A%5BTitle%2FAbstract%5D%29%29+OR+%28n3+Oil%2A%5BTitle%2FAbstract%5D%29%29+OR+%28n-3+PUFA%5BTitle%2FAbstract%5D%29%29+OR+%28n+3+PUFA%5BTitle%2FAbstract%5D%29%29+OR+%28n3+PUFA%5BTitle%2FAbstract%5D%29%29+OR+%28%22n-3+Fatty+Acid%2A%22%5BTitle%2FAbstract%5D%29%29+OR+%28%22n+3+Fatty+Acid%2A%22%5BTitle%2FAbstract%5D%29%29+OR+%28%22n3+Fatty+Acid%2A%22%5BTitle%2FAbstract%5D%29%29+OR+%28%22n-3+Polyunsaturated+Fatty+Acid%2A%22%5BTitle%2FAbstract%5D%29%29+OR+%28%22n+3+Polyunsaturated+Fatty+Acid%2A%22%5BTitle%2FAbstract%5D%29%29+OR+%28%22n3+Polyunsaturated+Fatty+Acid%2A%22%5BTitle%2FAbstract%5D%29%29+OR+%28%28%22Fish+Oil%2A%22%5BTitle%2FAbstract%5D%29+OR+%28%22Fish+Liver+Oil%2A%22%5BTitle%2FAbstract%5D%29%29&ac=no&sort=relevance) |
| #6 | Search: (("Fatty Acids, Omega-3"[Mesh]) OR "Fish Oils"[Mesh]) OR ((((((((((((((((Omega-3[Title/Abstract]) OR (Omega 3[Title/Abstract])) OR (Omega3[Title/Abstract])) OR (n-3 Oil*[Title/Abstract])) OR (n 3 Oil*[Title/Abstract])) OR (n3 Oil*[Title/Abstract])) OR (n-3 PUFA[Title/Abstract])) OR (n 3 PUFA[Title/Abstract])) OR (n3 PUFA[Title/Abstract])) OR ("n-3 Fatty Acid*"[Title/Abstract])) OR ("n 3 Fatty Acid*"[Title/Abstract])) OR ("n3 Fatty Acid*"[Title/Abstract])) OR ("n-3 Polyunsaturated Fatty Acid*"[Title/Abstract])) OR ("n 3 Polyunsaturated Fatty Acid*"[Title/Abstract])) OR ("n3 Polyunsaturated Fatty Acid*"[Title/Abstract])) OR (("Fish Oil*"[Title/Abstract]) OR ("Fish Liver Oil*"[Title/Abstract]))) | [45,268](https://pubmed.ncbi.nlm.nih.gov/?term=%28%28%22Fatty+Acids%2C+Omega-3%22%5BMesh%5D%29+OR+%22Fish+Oils%22%5BMesh%5D%29+OR+%28%28%28%28%28%28%28%28%28%28%28%28%28%28%28%28Omega-3%5BTitle%2FAbstract%5D%29+OR+%28Omega+3%5BTitle%2FAbstract%5D%29%29+OR+%28Omega3%5BTitle%2FAbstract%5D%29%29+OR+%28n-3+Oil%2A%5BTitle%2FAbstract%5D%29%29+OR+%28n+3+Oil%2A%5BTitle%2FAbstract%5D%29%29+OR+%28n3+Oil%2A%5BTitle%2FAbstract%5D%29%29+OR+%28n-3+PUFA%5BTitle%2FAbstract%5D%29%29+OR+%28n+3+PUFA%5BTitle%2FAbstract%5D%29%29+OR+%28n3+PUFA%5BTitle%2FAbstract%5D%29%29+OR+%28%22n-3+Fatty+Acid%2A%22%5BTitle%2FAbstract%5D%29%29+OR+%28%22n+3+Fatty+Acid%2A%22%5BTitle%2FAbstract%5D%29%29+OR+%28%22n3+Fatty+Acid%2A%22%5BTitle%2FAbstract%5D%29%29+OR+%28%22n-3+Polyunsaturated+Fatty+Acid%2A%22%5BTitle%2FAbstract%5D%29%29+OR+%28%22n+3+Polyunsaturated+Fatty+Acid%2A%22%5BTitle%2FAbstract%5D%29%29+OR+%28%22n3+Polyunsaturated+Fatty+Acid%2A%22%5BTitle%2FAbstract%5D%29%29+OR+%28%28%22Fish+Oil%2A%22%5BTitle%2FAbstract%5D%29+OR+%28%22Fish+Liver+Oil%2A%22%5BTitle%2FAbstract%5D%29%29%29&ac=no&sort=relevance) |

| #7 | Search: (("Breast Neoplasms"[Mesh]) OR (((((((((((((((((("Breast Neoplasm*"[Title/Abstract]) OR ("Breast Tumor*"[Title/Abstract])) OR ("Breast Cancer*"[Title/Abstract])) OR ("Breast Carcinoma*"[Title/Abstract])) OR ("Breast Neoplasia*"[Title/Abstract])) OR ("Breast Malignanc*"[Title/Abstract])) OR ("Mammary Cancer*"[Title/Abstract])) OR ("Cancer of Breast"[Title/Abstract])) OR ("Cancer of the Breast"[Title/Abstract])) OR ("Neoplasia of Breast"[Title/Abstract])) OR ("Neoplasia of the Breast"[Title/Abstract])) OR ("Breast Malignant Tumor*"[Title/Abstract])) OR ("Breast Malignant Neoplasm*"[Title/Abstract])) OR ("Malignant Tumor of Breast"[Title/Abstract])) OR ("Malignant Neoplasm of Breast"[Title/Abstract])) OR ("Human Mammary Carcinoma*"[Title/Abstract])) OR ("Human Mammary Neoplasm*"[Title/Abstract])) OR ("Human Mammary Cancer*"[Title/Abstract]))) AND ((("Fatty Acids, Omega-3"[Mesh]) OR "Fish Oils"[Mesh]) OR ((((((((((((((((Omega-3[Title/Abstract]) OR (Omega 3[Title/Abstract])) OR (Omega3[Title/Abstract])) OR (n-3 Oil*[Title/Abstract])) OR (n 3 Oil*[Title/Abstract])) OR (n3 Oil*[Title/Abstract])) OR (n-3 PUFA[Title/Abstract])) OR (n 3 PUFA[Title/Abstract])) OR (n3 PUFA[Title/Abstract])) OR ("n-3 Fatty Acid*"[Title/Abstract])) OR ("n 3 Fatty Acid*"[Title/Abstract])) OR ("n3 Fatty Acid*"[Title/Abstract])) OR ("n-3 Polyunsaturated Fatty Acid*"[Title/Abstract])) OR ("n 3 Polyunsaturated Fatty Acid*"[Title/Abstract])) OR ("n3 Polyunsaturated Fatty Acid*"[Title/Abstract])) OR (("Fish Oil*"[Title/Abstract]) OR ("Fish Liver Oil*"[Title/Abstract])))) | [730](https://pubmed.ncbi.nlm.nih.gov/?term=%28%28%22Breast+Neoplasms%22%5BMesh%5D%29+OR+%28%28%28%28%28%28%28%28%28%28%28%28%28%28%28%28%28%28%22Breast+Neoplasm%2A%22%5BTitle%2FAbstract%5D%29+OR+%28%22Breast+Tumor%2A%22%5BTitle%2FAbstract%5D%29%29+OR+%28%22Breast+Cancer%2A%22%5BTitle%2FAbstract%5D%29%29+OR+%28%22Breast+Carcinoma%2A%22%5BTitle%2FAbstract%5D%29%29+OR+%28%22Breast+Neoplasia%2A%22%5BTitle%2FAbstract%5D%29%29+OR+%28%22Breast+Malignanc%2A%22%5BTitle%2FAbstract%5D%29%29+OR+%28%22Mammary+Cancer%2A%22%5BTitle%2FAbstract%5D%29%29+OR+%28%22Cancer+of+Breast%22%5BTitle%2FAbstract%5D%29%29+OR+%28%22Cancer+of+the+Breast%22%5BTitle%2FAbstract%5D%29%29+OR+%28%22Neoplasia+of+Breast%22%5BTitle%2FAbstract%5D%29%29+OR+%28%22Neoplasia+of+the+Breast%22%5BTitle%2FAbstract%5D%29%29+OR+%28%22Breast+Malignant+Tumor%2A%22%5BTitle%2FAbstract%5D%29%29+OR+%28%22Breast+Malignant+Neoplasm%2A%22%5BTitle%2FAbstract%5D%29%29+OR+%28%22Malignant+Tumor+of+Breast%22%5BTitle%2FAbstract%5D%29%29+OR+%28%22Malignant+Neoplasm+of+Breast%22%5BTitle%2FAbstract%5D%29%29+OR+%28%22Human+Mammary+Carcinoma%2A%22%5BTitle%2FAbstract%5D%29%29+OR+%28%22Human+Mammary+Neoplasm%2A%22%5BTitle%2FAbstract%5D%29%29+OR+%28%22Human+Mammary+Cancer%2A%22%5BTitle%2FAbstract%5D%29%29%29+AND+%28%28%28%22Fatty+Acids%2C+Omega-3%22%5BMesh%5D%29+OR+%22Fish+Oils%22%5BMesh%5D%29+OR+%28%28%28%28%28%28%28%28%28%28%28%28%28%28%28%28Omega-3%5BTitle%2FAbstract%5D%29+OR+%28Omega+3%5BTitle%2FAbstract%5D%29%29+OR+%28Omega3%5BTitle%2FAbstract%5D%29%29+OR+%28n-3+Oil%2A%5BTitle%2FAbstract%5D%29%29+OR+%28n+3+Oil%2A%5BTitle%2FAbstract%5D%29%29+OR+%28n3+Oil%2A%5BTitle%2FAbstract%5D%29%29+OR+%28n-3+PUFA%5BTitle%2FAbstract%5D%29%29+OR+%28n+3+PUFA%5BTitle%2FAbstract%5D%29%29+OR+%28n3+PUFA%5BTitle%2FAbstract%5D%29%29+OR+%28%22n-3+Fatty+Acid%2A%22%5BTitle%2FAbstract%5D%29%29+OR+%28%22n+3+Fatty+Acid%2A%22%5BTitle%2FAbstract%5D%29%29+OR+%28%22n3+Fatty+Acid%2A%22%5BTitle%2FAbstract%5D%29%29+OR+%28%22n-3+Polyunsaturated+Fatty+Acid%2A%22%5BTitle%2FAbstract%5D%29%29+OR+%28%22n+3+Polyunsaturated+Fatty+Acid%2A%22%5BTitle%2FAbstract%5D%29%25) |
| --- | --- | --- |

**Table S2**. Characteristics of included studies and their risk of bias

| 1) Bjørklund G (2015) | | |
| --- | --- | --- |
| Method | Clinical trial, single arm | |
| Participants | 32 typical patients with breast cancer, aged 32–81 years of age and classified as high risk because of tumor spread to the lymph nodes in the axilla | |
| Interventions | The patients received a cocktail (consisting of vitamin C (2850 mg/day), vitamin E (2500 IU/day), beta-carotene (32.5 IU/day), Se (387 mg/day), various  other vitamins and essential trace elements, essential fatty acids (1.2 g gamma-linolenic acid/day and 3.5 g omega-3 PUFAs/day), and coenzyme Q10 (CoQ10, 90 mg/day)) But  later the protocol was changed, with reduction of the Se intake and more coenzyme Q10 than when the study was started | |
| Outcome | The main investigator died and the study outcome were reported briefly after 18 months. | |
| Risk of bias | Authors’ judgment | Support for judgment |
| Random sequence generation (Selection bias) | High risk | There was only one intervention group and no control group |
| Allocation concealment  (Selection bias) | High risk | There was only one intervention group and no control group |
| Blinding of participants  and personnel  (Performance bias) | High risk | There was only one intervention group and no control group |
| Blinding of outcome assessor (Detection bias) | High risk | There was only one intervention group and no control group |
| Incomplete outcome data  (Attrition bias) | High risk | There is no information |
| Selective reporting  (Reporting bias) | High risk | All outcomes were not reported |
| 2) Martínez N et al. (2018) | | |
| Method | Clinical trial, single arm | |
| Participants | forty-nine post- menopausal women with histopathological diagnosis of AJCC Stage 0-IIIA were entered at the trial and They should have a history of more than 12 months from their initial surgery for  breast cancer, at least 6 months since last chemotherapy,  with no evidence of disease | |
| Interventions | all enrolled patients took three capsules of PureVida™ contain 460 mg of fish  oil (EPA and DHA), 12.5 mg of natural hydroxytyrosol and 50 mg extract of curcumin  (47.5 mg curcuminoids) per day for 30 days (two in the morning during breakfast and one in the evening during dinner). | |
| Outcome | At 30 and 60 days two blood draws within 5 ± 2 days of each other were obtained. CRP, IL-6, SAA, IFNγ, TNFα, IL-10, IL-15, TGFβ, IGF-1, total cholesterol, HDL, LDL and triglycerides were measured in each sample.  The Brief Pain Intensity Score (BPI-SF) were asked to complete at baseline and at week 2 and 4 during therapy using BPI-SF questioner. | |
| Risk of bias | Authors’ judgment | Support for judgment |
| Random sequence generation (Selection bias) | High risk | There was only one intervention group and no control group |
| Allocation concealment  (Selection bias) | High risk | There was only one intervention group and no control group |
| Blinding of participants  and personnel  (Performance bias) | High risk | There was only one intervention group and no control group |
| Blinding of outcome assessor (Detection bias) | High risk | There was only one intervention group and no control group |
| Incomplete outcome data  (Attrition bias) | Low risk | Three of these did not start the treatment and were excluded, one stopped treatment due to an unplanned surgery, and 45  patients completed treatment as established per protocol |
| Selective reporting  (Reporting bias) | Low risk | All outcomes were reported |
| 3) Shen Sh et al (2018) | | |
| Method | Post hoc analysis of SWOG S0927 (Clinical trials).  gov NCT01385137) | |
| Participants | Two hundred and forty nine Post-menopausal women with stage I–III breast cancer  taking an aromatase inhibitor (AI) for ≥ 90 days were entered at the trial  Patients were categorized into BMI < 30 kg/m2 (non-obese) or ≥ 30 kg/m2 (obese).  Non-obese= 139 (56%) obese= 110 (44%) | |
| Interventions | Subjects were randomized to O3-FA (3.3 g per day; 560 mg eicosapentanoic acid plus docosahexaenoic acid in a 40:20 ratio) or placebo (soybean-corn oil blend) for 24 weeks. | |
| Outcome | The BPI-Short Form, a 14-item questionnaire for the assessment of cancer pain, was administered at baseline, 6, 12, and 24 weeks.  The Global Ratings of Change questionnaire regarding joint pain and stiffness was assessed at 6, 12, and 24 weeks.  Three other scales were also used to assess patients’ symptoms at the same time points, including the Modified Score for the Assessment and Quantification of Chronic Rheumatoid Affections of the Hands (M-SACRAH), the Western Ontario and McMaster Universities Osteoarthritis Index (WOMAC), and the Functional Assessment of Cancer  Therapy–Endocrine Symptoms (FACT-ES).  Fasting serum and Lipid profile were collected at baseline, 12, and 24 weeks.  BPI-SF questioner, The Global Ratings of Change questionnaire, M-SACRAH scale, WOMAC index, FACT-ES scale were used for data collecting. | |
| Risk of bias | Authors’ judgment | Support for judgment |
| Random sequence generation (Selection bias) | unclear risk | The randomization method was not described. |
| Allocation concealment  (Selection bias) | High risk | No specific information regarding allocation concealment has been given |
| Blinding of participants  and personnel  (Performance bias) | unclear risk | No specific information regarding participants and personnel blinding has been given |
| Blinding of outcome assessor (Detection bias) | unclear risk | No specific information regarding Blinding of outcome assessor has been given |
| Incomplete outcome data  (Attrition bias) | Low risk | No loss |
| Selective reporting  (Reporting bias) | Low risk | All outcomes were reported |
| 4) Darwito D et al (2019) | | |
| Method | RCT | |
| Participants | forty eight Patients confirmed diagnosis of breast cancer, locally advanced stage IIIB breast cancer, ductal invasive, age 25-60 years of age, who received neoadjuvant chemotherapy CAF were assigned to receive interventions. | |
| Interventions | All participants received standard neoadjuvant CAF chemotherapy (cyclophosphamide 600 mg/m2, doxorubicin 60 mg/m2, and 5-FU 600 mg/m2 for 3 cycles ((for 51 days).  Intervention group: 24 participants received an oil-fish capsule containing 1 g Omega-3 fatty acids per day  Control group: 24 participants received Placebo tablets similar to oil-fish capsule | |
| Outcome | Expression levels of *Ki-67* and *VEGF* were semi-quantified analyzed  Follow-up visits were structurally scheduled according to the hospital guidelines, Progression-free survival (PFS) and overall survival (OS) were determined to start from the time elapsed between diagnosis to any evidence of tumor progression and death from any cause, | |
| Risk of bias | Authors’ judgment | Support for judgment |
| Random sequence generation (Selection bias) | Low risk | Randomization was performed by assigning random numbers according to the random number tables to the intervention or control group. |
| Allocation concealment  (Selection bias) | High risk | No only allocation concealment was used |
| Blinding of participants  and personnel  (Performance bias) | unclear risk | No specific information regarding Blinding of participants and personnel has been given |
| Blinding of outcome assessor (Detection bias) | unclear risk | No specific information regarding participants and personnel blinding has been given |
| Incomplete outcome data  (Attrition bias) | Low risk | No loss. 3 patients suffered from diarrhea and the supplementation of Omega-3 was paused for 5-7 days. |
| Selective reporting  (Reporting bias) | Low risk | All outcomes were reported |
| 5) Paixão EMDS et al (2017) | | |
| Method | RCT | |
| Participants | Forty five patients with treatment-naïve between 18 and 70 years of age,  with mammographic image classification 4C or higher and only after positive biopsy confirmation for malignancy were divided in two groups including fish oil  group (n=23), Placebo group (n=22) | |
| Interventions | Intervention group:23 participants received 1g gel capsules contain fish oil 2 times daily for 30 days (with a total of 1.81 g of n-3 fatty acids per day)  Control group:22 participants received 1g gel capsules contain mineral oil similar to fish oil capsules  “The intervention lasted 30 days, immediately following the diagnosis and before the surgical procedure”. | |
| Outcome | Twelve hours fasted blood samples were collected for biochemical and immunological analyses at baseline and at the end of the intervention period.  Biochemical analysis included: serum glucose, total cholesterol, high- and low density  Lipoprotein cholesterol and triglycerides, complete blood count, albumin and high sensitivity C-reactive protein.  Immunological parameters included: peripheral blood mononuclear CD4+ e CD8+ lymphocyte cell counts, plasma cytokines(IL-6, IL-1β and TNF-α) and PGE2 .  Plasma phospholipid fatty acid profile.  Dietary intake was evaluated by 24-h recall method, two at baseline and two at the end of intervention.  Body weight, height and body composition analyses were performed at baseline and at the end of the intervention period.  ELISA method (EIA kit), phospholipids were separated by thin layer chromatography with solvent system hexane: diethyl ether: acetic acid (80:20:2 v/v/v). | |
| Risk of bias | Authors’ judgment | Support for judgment |
| Random sequence generation (Selection bias) | Low risk | The randomization was done beforehand and performed by manual raffling the blocks of ten sequential numbers with five chances of being raffled to one of the two groups. |
| Allocation concealment  (Selection bias) | Low risk | The supplements were supplied in white plastic bottles and added subtle fish oil smell to the bottles of mineral oil (placebo), thus, all patients thought they were receiving fish oil capsules. |
| Blinding of participants  and personnel  (Performance bias) | Low risk | A laboratory technician not involved in the research performed the randomization |
| Blinding of outcome assessor (Detection bias) | Low risk | This study was a double blinded controlled trial |
| Incomplete outcome data  (Attrition bias) | Low risk | eight of the participants discontinued the study due to supplement intolerance (n = 2) and to change to neoadjuvant chemotherapy as primary treatment (n = 6).Thirty seven patients completed the study, of whom 18  were supplemented with fish oil and 19 with placebo |
| Selective reporting  (Reporting bias) | Low risk | All outcomes were reported |
| 6) F DelaRosa-Oliva et al (2017) | | |
| Method | RCT | |
| Participants | Forty four LABC (locally advanced breast cancer), clinical stage IIA-III B, patients with no differences in age between the supplemented and the placebo groups (51.6y vs. 49.1y, *p*=0.4) were divided in two groups including Supplementation (n=24), pelacebo group (n=24) | |
| Interventions | Intervention group:22 participants received supplement 2.4g/d of EPA and DHA (1.6 g EPA and 0.8 g DHA, ratio 2:1) during the 6 months  Placebo group:22 participants received placebo similar to supplement | |
| Outcome | assessment were performed before each CT, and also blood total leukocytes, and percentage lymphocytes and monocytes | |
| Risk of bias | Authors’ judgment | Support for judgment |
| Random sequence generation (Selection bias) | Unclear risk | This study was a poster |
| Allocation concealment  (Selection bias) | Unclear risk | This study was a poster |
| Blinding of participants  and personnel  (Performance bias) | Unclear risk | This study was a poster |
| Blinding of outcome assessor (Detection bias) | Unclear risk | This study was a poster |
| Incomplete outcome data  (Attrition bias) | Unclear risk | This study was a poster |
| Selective reporting  (Reporting bias) | Low risk | All outcomes were reported |

| 7) DelaRosa-Oliva F et al (2019) | | |
| --- | --- | --- |
| Method | RCT | |
| Participants | Fifty three participants with LABC (locally advanced breast cancer), clinical stage IIA-III B, during the six months of NeoCT (neoadjuvant chemotherapy) between 18-80 years of age divided in two groups including PUFA Ω-3 group(n=27), placebo group(n=26) | |
| Interventions | Intervention group: 27 participants received an oral supplement with PUFA Ω-3 (in a 2:1ratio of DHA/EPA) derived from fish oil in gel capsules, daily dosed at 2.4 g (four capsules) during the six months of chemotherapy.  Placebo group:26 received placebo(sun flower) similar gel capsule | |
| Outcome | Body composition evaluation: Weight and body composition (body fat mass, fat-free mass, skeletal muscle, body mass index [BMI])  Metabolic evaluation: Serum glucose, lipid profile (total cholesterol, HDL and LDL cholesterol and triglycerides), insulin, glycated hemoglobin (HbA1c), liver  function tests, urea and creatinine, Insulin resistance (IR)  Measurements and blood samples were obtained at the beginning of the study  ,three and six months after therapy | |
| Risk of bias | Authors’ judgment | Support for judgment |
| Random sequence generation (Selection bias) | Unclear risk | The randomization method was not described |
| Allocation concealment  (Selection bias) | High risk | No allocation concealment method was used |
| Blinding of participants  and personnel  (Performance bias) | Low risk | Capsules were identical in appearance |
| Blinding of outcome assessor (Detection bias) | Low risk | Capsules were identical in appearance |
| Incomplete outcome data  (Attrition bias) | Low risk | One of the participants discontinued treatment |
| Selective reporting  (Reporting bias) | Low risk | All outcomes were recorded |

| 8) Zachary B et al (2020) | | |
| --- | --- | --- |
| Method | RCT(brief report) | |
| Participants | One hundred postmenopausal women with estrogen receptor-positive breast cancer undergoing surgery were divided in two groups including test and placebo | |
| Interventions | Intervention group: omega-3 FFAs, 2700 mg by mouth twice daily + letrozole during the 21-30 days prior to surgery  control group: letrozole 2.5 mg by mouth/daily during the 21-30 days prior to surgery | |
| Outcome | A follow-up clinical exam was performed by a surgeon 2 weeks after surgery determine SSHCs and SSIs | |
| Risk of bias | Authors’ judgment | Support for judgment |
| Random sequence generation (Selection bias) | Unclear risk | The randomization method was not described. |
| Allocation concealment  (Selection bias) | High risk | No allocation concealment method was used |
| Blinding of participants  and personnel  (Performance bias) | High risk | No blinding of participants was used |
| Blinding of outcome assessor (Detection bias) | High risk | No blinding of outcome assessor was used |
| Incomplete outcome data  (Attrition bias) | High risk | 69 participants didn’t complete the study |
| Selective reporting  (Reporting bias) | Low risk | All out comes were reported |
| 9) Bougnoux P et al (2009) | | |
| Method | Open-labelled phase II trial | |
| Participants | Twenty five Breast cancer patients between 25-75 years of age with visceral metastases | |
| Interventions | Twenty five participant received nine capsules of DHASCO daily (representing 200 mg *9=1800 mg DHA/day), as three capsules at each meal. | |
| Outcome | Complete clinical examination, biochemical analysis, electrocardiogram, cardiac sonography and pulmonary testing (spirometry, gas diffusion) were performed at baseline. Clinical examination and a biochemical analysis were repeated at day 1 of each cycle.  Assessment of tumor response through clinical and radiological  evaluations was performed every two cycles, and compared with  baseline  Time to progression (TTP) and overall survival (OS).  Plasma DHA. | |
| Risk of bias | Authors’ judgment | Support for judgment |
| Random sequence generation (Selection bias) | High risk | There was only one intervention group and no control group |
| Allocation concealment  (Selection bias) | High risk | There was only one intervention group and no control group |
| Blinding of participants  and personnel  (Performance bias) | High risk | There was an open-labelled study |
| Blinding of outcome assessor (Detection bias) | High risk | There was an open-labelled study |
| Incomplete outcome data  (Attrition bias) | Low risk | No loss |
| Selective reporting  (Reporting bias) | Low risk | All outcomes were reported |
| 10) Wanli Xu et al (2022) | | |
| Method | RCT | |
| Participants | Forty six participants with 6-24 months post-treatment for early-stage BC (stage I to IIIA) between 30-75 years of age enrolled in phase I | |
| Interventions | This study has two phase :  Phase I: forty six participants received a personalized meal plan was formulated by the research team members trained in nutrition immediately during 3 weeks  Phase II: thirty nine participants divided in two groups to high omega-3LC (n=24), low omega-3LC ( n=15) (wild-caught Alaskan salmon; high = 12 ounces/week, 2040 mg omega-3LC; low = 6ounces/week, 1020 mg | |
| Outcome | Pain, depressive symptoms, fatigue, sleep disturbance , Perceived stress, Salivary sample collection | |
| Result | BPI-SF questioner, CES-D scale, BFI scale, GSDS scale | |
| Risk of bias | Authors’ judgment | Support for judgment |
| Random sequence generation (Selection bias) | Low risk | Randomization was accomplished using an assignment schedule created using GraphPad Prism version 7.00 for Windows, GraphPad Software, La Jolla Cali- fornia USA |
| Allocation concealment  (Selection bias) | High risk | No allocation concealment method was used |
| Blinding of participants  and personnel  (Performance bias) | High risk | No Blinding of participants and personnel  was used |
| Blinding of outcome assessor (Detection bias) | High risk | No blinding of outcomes assessor was used |
| Incomplete outcome data  (Attrition bias) | Low risk | Discontinued phase I n=7(unresponsive=1, pre-exiting medical condition=5, car accident=1)  Discontinued phase II n=5(lost to follow up=4, mobility issues and unable to contact=1) |
| Selective reporting  (Reporting bias) | Low risk | All outcomes were reported |
| 11) Kleckner AS et al (2021) | | |
| Method | RCT(phase II) | |
| Participants | Eighty five participants with 4–36 months post-treatment (surgery, radiation,  and/or chemotherapy) for stage 0-III cancer who were 18 years old or older | |
| Interventions | “Participants were allocated 1:1:1 to one of three supplement groups: 6 g fish oil n=30, 6 g soybean oil (control)n=30, or 3 g of each daily for 6 weeks n=23” | |
| Outcome | Omega-3 fatty acids, omega-6 fatty acids, and cholesterol were quantified at baseline and post-intervention | |
| Measures | MFSI questioner, | |
| Risk of bias | Authors’ judgment | Support for judgment |
| Random sequence generation (Selection bias) | Low risk | Randomization was accomplished by random algorithm |
| Allocation concealment  (Selection bias) | High risk | No only Allocation concealment was used |
| Blinding of participants  and personnel  (Performance bias) | High risk | No only blinding of participants and personnel was used |
| Blinding of outcome assessor (Detection bias) | High risk | No only Blinding of outcome assessor was used |
| Incomplete outcome data  (Attrition bias) | Low risk | No loss |
| Selective reporting  (Reporting bias) | Low risk | All outcomes were reported |
